# Assessment of Umbilical Cord Mesenchymal Stem Cell Cultivation Using Fetal Bovine Serum or Platelet Lysate

**DOI:** 10.7759/cureus.78044

**Published:** 2025-01-27

**Authors:** Gamila A Attaelmanan, Hiba B Khalil

**Affiliations:** 1 Medical Laboratory Sciences/Hematology, Al Neelain University, Khartoum, SDN; 2 Hematology and Stem Cell Technology, Al Neelain Stem Cell Center, Khartoum, SDN

**Keywords:** cord blood, cultivation protocol, fetal bovine serum, flow cytometry, mesenchymal stem cell, platelet lysate, wharton’s jelly

## Abstract

Background: Wharton’s jelly (WJ) and umbilical cord blood (UCB) are considered marvelous sources of mesenchymal stem cells (MSCs) due to their availability, simply isolated without pain and ethical issues. Additionally, UC-MSCs are more primitive than MSCs isolated from adult sources, thus opening new possibilities for cell therapies.

Objective: In this study, we aimed to develop a simple, economical, and efficient xenogenic-free protocol for the isolation and expansion of UC-MSCs to be compliant with good manufacturing practice (GMP) guidelines.

Methods/design: In this work, we used the explant-partial enzymatic digestion technique for WJ-MSCs and Ficoll-Paque density centrifugation for CB-MSC isolation. Human platelet lysate (HPL) was used as a replacement for fetal bovine serum (FBS), which is used in most protocols.

Results: We observed that the explants-partial enzymatic digestion technique is an effective protocol for MSC isolation from WJ, and HPL is a safe, powerful, and cost-effective substitute for FBS in the MSC propagation. Among all tested conditions, 10% HPL demonstrated the best growth rate, highest viability count, and highest expression level of a cluster of differentiation markers (CD).

Conclusion: We concluded that WJ-MSCs are superior to UCB-MSCs in the matter of rapidity and homogeneity, and both of them expressed MSC-positive markers CD44 and CD73, suggesting that WJ is a source with greater potential for MSC isolation for clinical application.

## Introduction

Mesenchymal stem cells (MSCs) are pluripotent stromal cells found in many different types of human tissues. Recently, they have possessed huge promise in regenerative medicine due to their capability of self-renewal and differentiation into mesodermal, endodermal, and ectodermal lineages plus their ability to produce numerous trophic factors, which encourage cell survival and inhibit apoptosis, promote angiogenesis, and possess immunomodulatory, anti-inflammatory, and anti-oxidative properties [1]. In addition, MSCs have the ability to homing to injured sites, and they support and regulate hematopoiesis. These unique properties make MSCs of great interest in cell therapy and tissue engineering [2].

The umbilical cord (UC) is considered a golden source for MSC isolation due to its easy availability; it avoids invasive procedures and harm to the mother or the infant [3]. In addition to resolving the ethical and political concerns related to the use of embryonic stem cells, MSCs have been isolated from the various regions of the UC. However, several studies have displayed that the UC matrix “Wharton′s jelly” (WJ) is the top option for isolating MSCs for clinical applications in the future, as it possesses a 100% success rate of isolating MSCs, while UC blood (UCB) does not exceed 60% [4,5].

UC-MSCs have more primitive properties, a lower risk of genetic alterations and viral contamination [3], and a higher proliferation rate in vitro when compared with adult MSCs; they also can be expanded and cryopreserved for use in future therapy. These different physiological properties are likely due to their naïve status [6].

Owing to the discrepancy in MSC isolation and expansion protocols, the International Society of Cellular Therapy has recommended three basic criteria for the characterization of MSCs: MSCs should be plastic-adherent; when cultured under proper conditions, they must express specific markers such as CD105, CD90, and CD73 and lack CD45, CD34, CD14, or CD11b, CD79 alpha or CD19, and HLA-DR expression; and they must have the capability to differentiate into chondroblasts, osteoblasts, and adipocytes under stander differentiation conditions [7].

The use of MSCs in clinical applications depends on the selection of a proper primary isolation method, along with an efficient expansion protocol to obtain sufficient cell yield and maintain cellular quality as much as possible while avoiding adverse patient reactions [8,9]. In this regard, there are many different techniques to isolate UC-MSCs according to the site of isolation. The main technique to isolate MSCs from cord blood is density gradient purification, usually using Ficoll-Paque as a separation media, after which MSCs are isolated by their adherence to plastic in primary culture [10]. On the other hand, mainly there are two techniques used by researchers for MSC isolation from UC tissue “Wharton′s jelly": enzyme digestion and explants technique. Protocols based on digestion generally use enzymes to digest tissues and liberate cells from WJ, which then attach to the surface of culture vessels [11]. The explant protocol depends on the ability of MSCs to migrate from UC tissues to the surface of culture vessels [12]. Although the enzymatic technique is less time-consuming, it is also harmful to cells; it causes extracellular meshwork degradation, which then prevents MSCs from adhering to the surface of culture vessels, subsequently affecting the quality and quantity of isolated cells. Conversely, explant protocols are simpler and more cost-effective than enzymatic digestion protocols as they do not need incubation with enzymes [13].

Furthermore, previous studies have reported that the explant technique could harvest a less heterogeneous cell population with a high proliferation rate and cell viability in comparison with the enzymatic technique. Indeed the explant technique is better than the enzymatic digestion method, but the explant technique takes longer to obtain cells in the initial step of primary culture compared to the enzymatic method [13,14].

To overcome the disadvantages of enzymatic and explant techniques, we developed and optimized a combined enzymatic-explant protocol that balances efficiency and cell viability. Specifically, we used the trypsin enzyme alone for a short time as partial digestion, which reduces proteolytic stress and helps the release of cells from the tissue. While the presence of tissue pieces mimics the in vivo environment for the migrating cells by releasing growth factors and cytokines, in this protocol, we reduced the harmful effects and costs of complete and combined enzyme digestion techniques that were reported in previous research. Moreover, we reduced the time needed to release cells from tissue when using the explant technique.

Nowadays, fetal bovine serum (FBS) is considered the most common growth factor and attachment supplement used for culture media [15]. However, the regulatory authorities discouraged using of FBS for the ex-vivo expansion of MSCs [16] because they recognized that there is a great variation in growth factor activity between FBS batches and lots, due to the absence of standardization in FBS preparations, which leads to inconsistency in cell culture performance [17]. Additionally, FBS is associated with the transmission of prion or viral disease, as well as the generation of anti-FBS antibodies and possible causation of anaphylatoxin reaction [18]. Moreover, the high cost of FBS and ethical issues related to its manufacturing lead to the exploration of possible alternatives that promote MSC expansion for clinical application [19].

Lately, human platelet lysate (HPL) has been a more favorable human product and a competent choice as an alternative to FBS because it originates from humans in addition to its low cost, accessibility, and safety. Furthermore, HPL is rich in growth factors and bioactive molecules that are secreted from α-granules after platelet activation physically or physiologically. These contain coagulation factors, proteoglycans, protease inhibitors, adhesion molecules, epidermal growth factor (EGF), basic fibroblast-derived growth factor (bFGF), HGF, vascular endothelial growth factor, insulin-like growth factor-1 (IGF-1), vascular endothelial growth factor (VEGF), transforming growth factor-beta1 (TGF-β1), soluble CD40, interleukin (IL), intercellular adhesion molecule-1, vascular cell adhesion molecule-1, platelet-derived growth factor receptor AA (PDGF-AA), platelet-derived growth factor receptor AB (PDGF-AB), platelet-derived growth factor receptor BB (PDGF-BB), chemokine (C-C) ligand 5, and chemokine (C-X-C) ligand 1/2/319. All of these molecules stimulate cell proliferation and function in contrast with FBS [20-22].

There are many different procedures for platelet lysate preparation, with each lab designing its own procedure. Generally, HPL is obtained by activation of thrombocytes either physiologically by using collagen or calcium and/or thrombin or by mechanical rupture of platelets through freezing and thawing cycles [23,24]. Variations in the procedures of HPL preparation may influence the platelet granule disruption, which then could affect the concentration and quality of released growth factors [25]. Many previous studies showed that the proliferation rate and colony frequency of bone marrow (BM) MSCs (BM-MSCs) are significantly higher when using platelet derivatives generated by calcium chloride (CaCl_2_) activation compared with that produced by freezing/thawing [26]. Moreover, using CaCl_2_ enables the avoidance of using heparin, which is added to prevent culture media gelation when using a method like freezing-thawing. Heparin could affect MSC proliferation and cause hypersensitivity, and it is non-xeno-free. Similarly, optimal parameters of the freezing-thawing method, such as temperature and cycle number of the freezing and thawing, remain undefined [27,28]. On the other hand, Giraldo et al. noted that CaCl_2_ produced gross salt deposition and that calcium gluconate is the better substance to induce growth factors free release from calcium precipitation in the clot [29]. Consequently, we used calcium gluconate for platelet lysate preparation.

Choosing the ideal MSC source and establishing optimal isolation and expansion protocols are essential for achieving the success of MSC-based therapies. Therefore, the present study aims to provide a protocol that can improve the isolation and culturing process of UC-MSCs with minimal risk and lower cost. Furthermore, we aimed to assess and compare the properties of MSCs from UCB and UC tissue (WJ) under xenogeneic culture conditions.

## Materials and methods

This study was carried out at the stem cell culture laboratory, Faculty of Medical Laboratory Sciences, Al Neelain University, Khartoum, Sudan. A total of 19 UC samples were collected at vaginal delivery (n = 2) or cesarean section (n = 17) from healthy donors, with a mean age of 31 years and a mean gestation period of 38 weeks. The newborn's genders were nine males and 10 females. Ten samples were UC-WJ (52.6%), and nine were cord blood (47.4%). One BM sample from a normal donor was used as a control.

Approximately 40 mL of heparinized UCB and 12 cm of UC tissue were collected for MSC isolation, purification, and identification. The control BM sample was collected for the same cultivation procedures to check working media, FBS, and HPL. Platelet concentrate (PC) units for lysate preparation were collected and approved from the national blood bank (< two days store).

Signed and informed consent was obtained from each donor before sample collection with the aid of a structured questionnaire, and ethical approval was gained from the National Health Research Ethics Committee of the Ministry of Health-Directorate of Private Medical Facilities, Khartoum, Sudan. All methods were performed according to its guidelines and regulations.

Preparation for Aseptic Working Conditions

Before the protocol, the workbench, safety cabinet, and cultivation instruments were disinfected with 70% ethanol, and media supplements and reagents were thawed at room temperature or in a water bath at 37°C.

Preparation of Platelet Lysate

We obtained fresh PC units from a licensed and accredited national blood bank. Each unit was tested and confirmed negative screening for infectious diseases. Preparation of HPL was performed under aseptic conditions inside the laminar flow using calcium gluconate. Platelets in PC units were washed and activated with 100 µL of 10% calcium gluconate (0.23 mmol Ca++/mL) per 1 mL of PC and then incubated for 30 minutes. After clotting, the tube was centrifuged, and the supernatant was collected (human platelet lysate). Filtration was done by using 0.2 µm filters to take out any cell debris and clot remnants and to ensure purity and sterility. Additionally, HPL was incubated under UV light for 30 minutes for further sterilization and inactivation of viruses. The prepared HPL was aliquoted and stored at -20°C until use for media supplementation.

Preparation of Culture Media

The products used were a low-glucose Dulbecco’s Modified Eagle’s Medium (DMEM) “powdered medium” from Gibco by Life Technologies (Chicago, IL; REF31600-083/LOT 1850559).

Preparation of the Powder Medium

Approximatey 500 mL of purified water was added to 5 g of the powder medium with gentle stirring at room temperature. Approimately 1.85 g of sodium bicarbonate was then added and mixed until dissolved. A pH value of 7.4 was obtained with a pH meter, and the media was then filtered using a 0.2 µm filter into a sterile container and stored at 4°C until use.

Preparation of Working Media

Specifically, 1% antibiotic-antimycotic (penicillin/streptomycin and amphotericin B) and 1% glutamine (all from Biowest USA, Inc., Lakewood Ranch, FL) were added to the working media. Different concentrations of either HPL or FBS were then added to this medium, G1 (three cultured flasks with FBS in 5%, 7%, and 10% concentrations) and G2 (same, but with HPL).

Protocol of MSC Isolation-MSC-UB Tissue (WJ)

**Figure 1:**
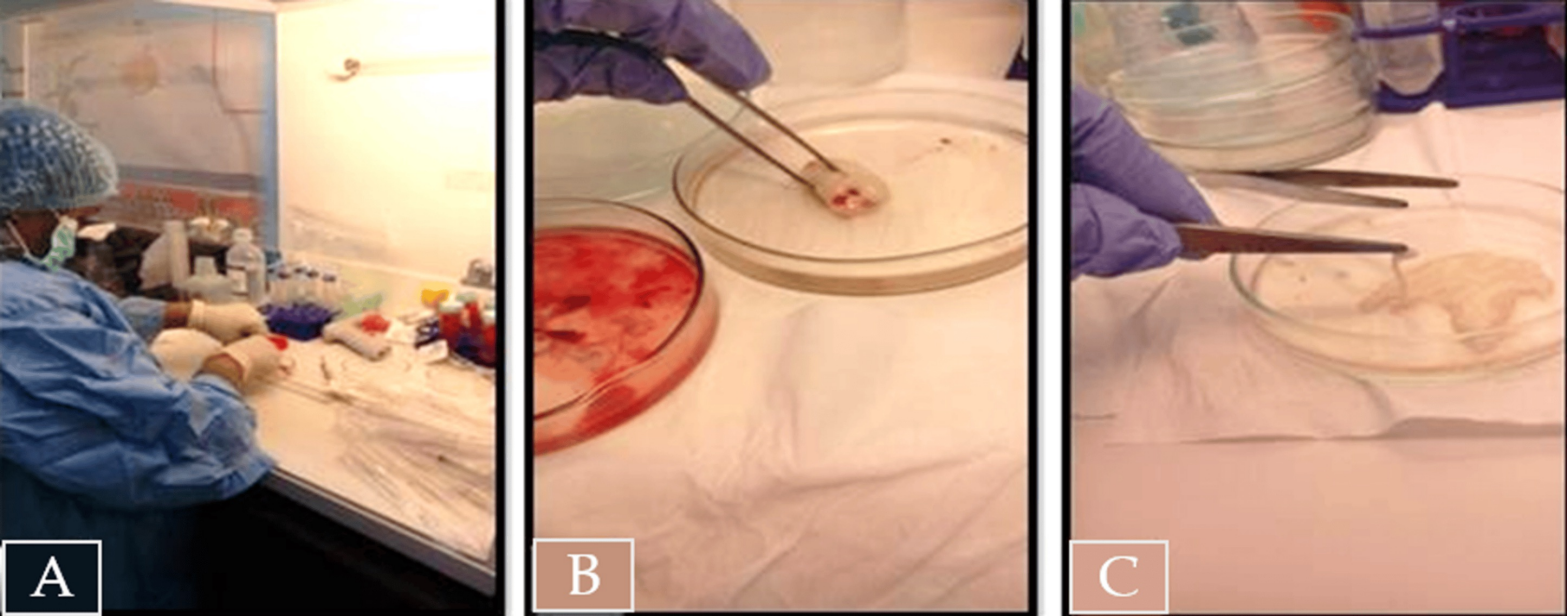
Preparation of umbilical cord tissue for WJ-MSC isolation A - Cutting of the umbilical cord tissue to a 2 cm piece. B - 2 cm of the umbilical cord tissue piece after washing with PBS and removing blood clots. C - Incising of cord tissue pieces longitudinally and removing blood vessels.

Within the biological safety cabinet, the surfaces of each umbilical cord were rinsed with a sterile solution of phosphate-buffered saline (PBS) (Gibco by Life Technology, Carlsbad, CA) and 1% penicillin/streptomycin to remove blood as much as possible. Each cord was cut up into 2 cm pieces, and blood vessels were removed by longitudinal cord incision (Figures 1A-1C). Pieces were then washed in PBS to remove the remaining blood. PBS was then replaced with 3-5 mL of 0.25% trypsin-EDTA solution (Gibco by Life Technology). Each 2 cm piece was then cut into small pieces 1-2 mm in size and then incubated with a trypsin solution in 5% CO_2_ for 30 minutes for partial digestion. Equal volumes of a culture media supplemented with 10% FBS or 10% HPL (Gibco by Life Technology) were then added to partially digested tissue pieces for trypsin activity neutralization. Then, the samples were transferred to 50 mL falcon tubes and centrifuged for 10 minutes, with any supernatant carefully removed.

Protocol of MSC Isolation 

Cord blood-MSCs and BM-MSCs: Each sample was diluted with PBS 1:1 and gently mixed. Ficoll-Paque (density 1.077 g/mL) (Sigma-Aldrich, St. Louis, MO) was placed in a 50 mL sterile tube and slowly topped with diluted cord blood or a BM sample at a 1:1 ratio, forming separate layers. Then, the samples were centrifuged at x500 g for 30 min at RT, and the top layer was discarded without disruption to the lower mononuclear cell (MNC) layer. MNC was then collected by a circular motion and placed into a suspension of 10 mL media, with cells washed at x400 g for seven minutes. After that, the supernatant was discarded, and the cell pellet was suspended in 10 mL of the culture media. Throughout this protocol, products were maintained at a temperature of 2-8°C.

Cultivation and Expansion of WJ-MSCs

About 15-20 pieces of partially digested tissue were plated in 75 cm^2^ tissue culture flasks, and then 10 mL of culture media with different concentrations of HPL or FBS was added and then incubated in 5% CO_2_ at 37°C and left undisturbed for periods of two to three days to allow plastic adherence of tissue pieces. Subsequently, the culture media was changed every three days. Outgrowths of cells from the explants were observed through an inverted microscope (Nikon TS100 inverted phase contrast microscope) daily.

Tissue pieces were removed after 10 days; the attached cells from explants were seeded to reach 80-90% confluence (P0). At 19 days, adherent cells were dissociated using 3 mL 0.25% EDTA trypsin and incubated in 5% CO_2_ at 37°C for three minutes. An equal volume of culture media containing 10% FBS or HPL was then added to the flask to stop trypsinization, and detached cells were transferred into falcon tubes and centrifuged at 1,000 rpm for five minutes, with the supernatant discarded. Approximately 1 mL of culture media was then added to the cell pellet for identification and viability counting.

Cultivation and Expansion - MSCs of CB and BM

The protocol for the culture of cord blood or BM was undertaken in much the same way; the MNC was seeded by 10 mL media with concentrations of 5%, 7%, and 10% FBS or HPL in 75 cm_2_ culture flasks and incubated in 5% CO_2_ at 37°C to reach 80-90% confluence (P0). After 24 hours, non-adherent cells were removed, and the culture media was replaced with a fresh one, cell growth was observed daily, and the culture media was changed every three days. Adherent cells were dissociated, and viable cells were counted using the same procedure applied to WJ-MSCs.

Characterization of MSCs - Trypan Blue for MSC Viability

Specifically, 10 µL of cell suspension was added to 90 µL of trypan blue (Gibco by Life Technology) in an Eppendorf tube and mixed well (DF 1:10). All viable and live cells with membrane integrity appeared clear and shiny as they had not taken up the dye, while dead cells were stained blue. The hemocytometer was prepared according to the manufacturer’s instructions with a mixture of cells in trypan blue, with an incubation period not exceeding five minutes to avoid cell toxicity. Viable and shiny (unstained) cells were counted under a microscope at ×10 of the total white blood cells count area and calculated (cell/mL) using the following formula: Viable cell count = Average of 4 squares × 0.01 (Chamber width × length × depth) × Dilution factor of trypan blue added × Volume of cells in suspension.

Giemsa Stain

Culture media was removed, and the culture surface was washed with PBS. Adherent cells were then fixed with methanol and left to dry. Once dried, adherent cells were then covered with fresh Giemsa stain for 30 min, and the flask was washed with PBS to remove excess stain. Adherent cell morphology was then evaluated under an inverted microscope.

MSC Markers by Flow Cytometer

Surface marker expressions for FBS and HPL expanded MSCs were analyzed by flow cytometer (Epics XL-MCL; Beckman Coulter, Brea, CA), using monoclonal antibodies/PE-conjugated CD34 and CD44 and FITC-conjugated for CD45 and CD73 (Immunostep, Salamanca, Spain). This protocol involved 20 µL of antibody added to 100 µL of MSC (max 1 × 10 cells/mL) in a 12x75 mm-labeled cytometer tube and gently mixed. The mixture was then incubated for 15 minutes in the dark at room temperature (20-25°C), centrifuged at 150 x g for five minutes, and the supernatant carefully aspirated to avoid disruption to the cell pellet. Then, 2 mL of wash solution was added, and the suspension was centrifuged at 540 x g for five minutes. Following supernatant aspiration, cells were then re-suspended in 0.3 mL of flow cytometry solution with controls prepared for every marker. Cells were then analyzed by a flow cytometer.

Statistical Analysis

Data were presented as means ± standard deviation. One-way analysis of variance was used for statistical comparisons. All p-values < 0.05 were considered statistically significant. GraphPad Prism software (version 7, GraphPad Software, San Diego, CA) was used to create the viability test figure.

## Results

Morphological characterization

BM-Derived MSCs (Control)

**Figure 2:**
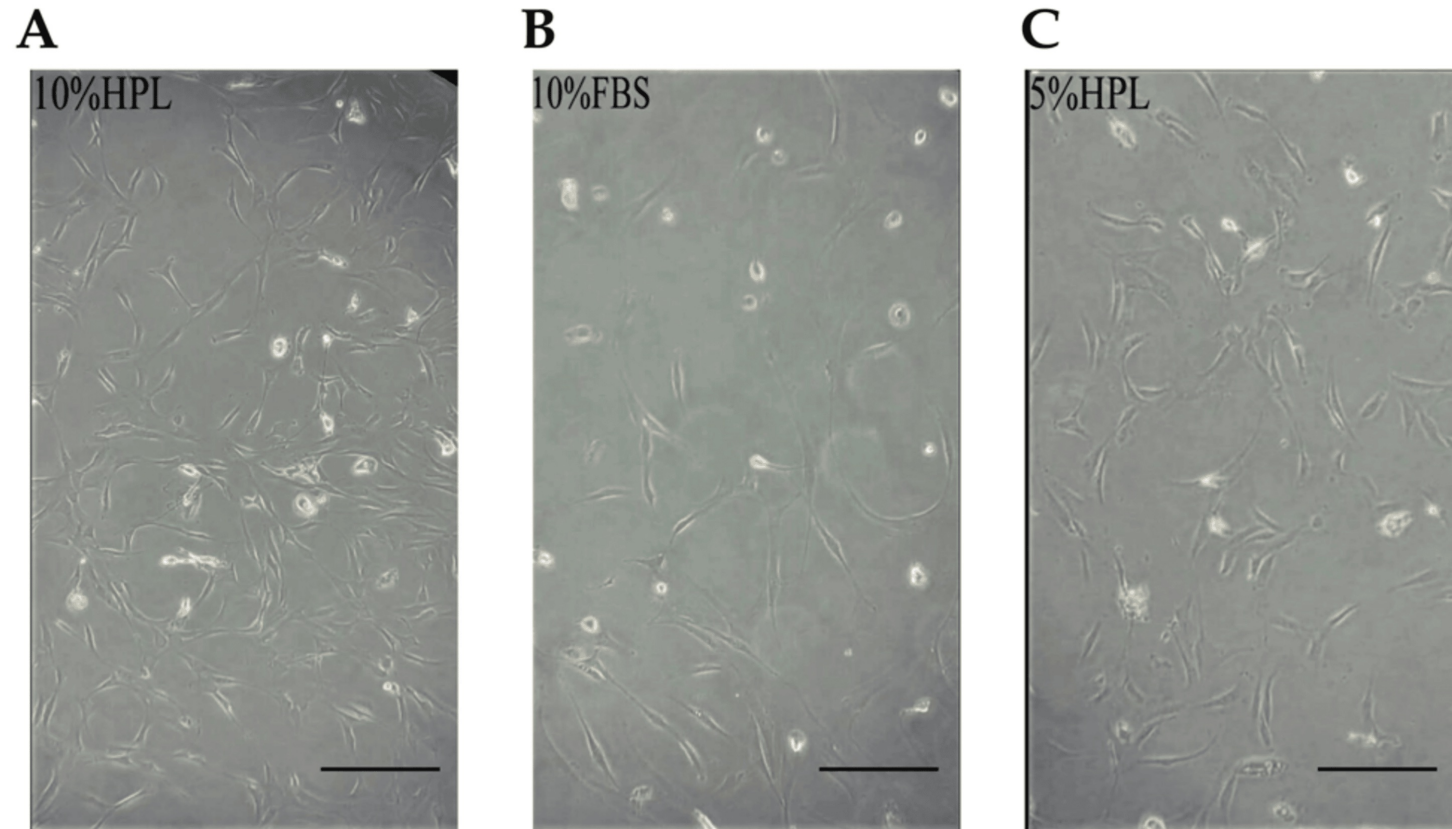
Bone marrow-derived MSCs HPL and FBS effect on cellular morphology and proliferation of human BM-MSCs on day nine. (A) - 10% HPL exhibited a high number of shiny viable cells in a short time when compared with 10% FBS (B) and 5% HPL (C), which showed almost identical proliferation capacity but 5% HPL displayed more shiny cells. Scale bar = 100 µm

Expanded BM-MSCs in media supplemented with 5% HPL or 10% FBS or 10% HPL are described by a long, shiny spindle (fibroblast-like) shape. In contrast, MSC expansion in 10% HPL produced the shiniest (most viable) cells in the shortest proliferation time (<10 days to reach 80-90% confluence). The proliferation time of MSCs in 5% HPL was almost identical to 10% FBS but with more shiny cells (Figure 2).

WJ-Derived MSCs

**Figure 3:**
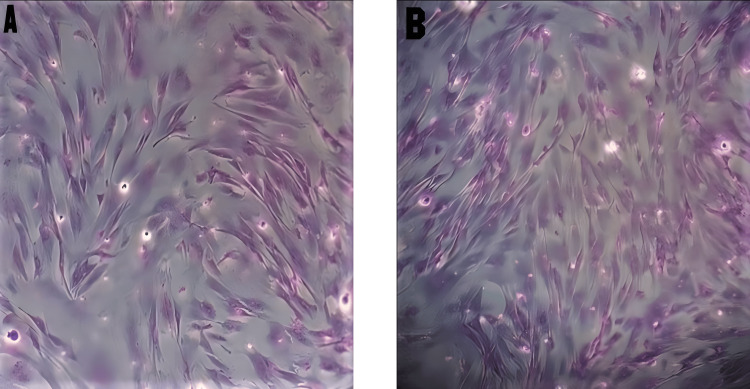
Giemsa stain of CFU-F in 10% FBS and 10% HPL at P0 day 19 A - 10% FBS showed loosely connected colonies with few cells. B - 10% HPL presented densely packed colonies with a high number of cells. Cells in the FBS medium showed a more flattened and irregular morphology than cells in HPL. Magnification 200×.

**Figure 4:**
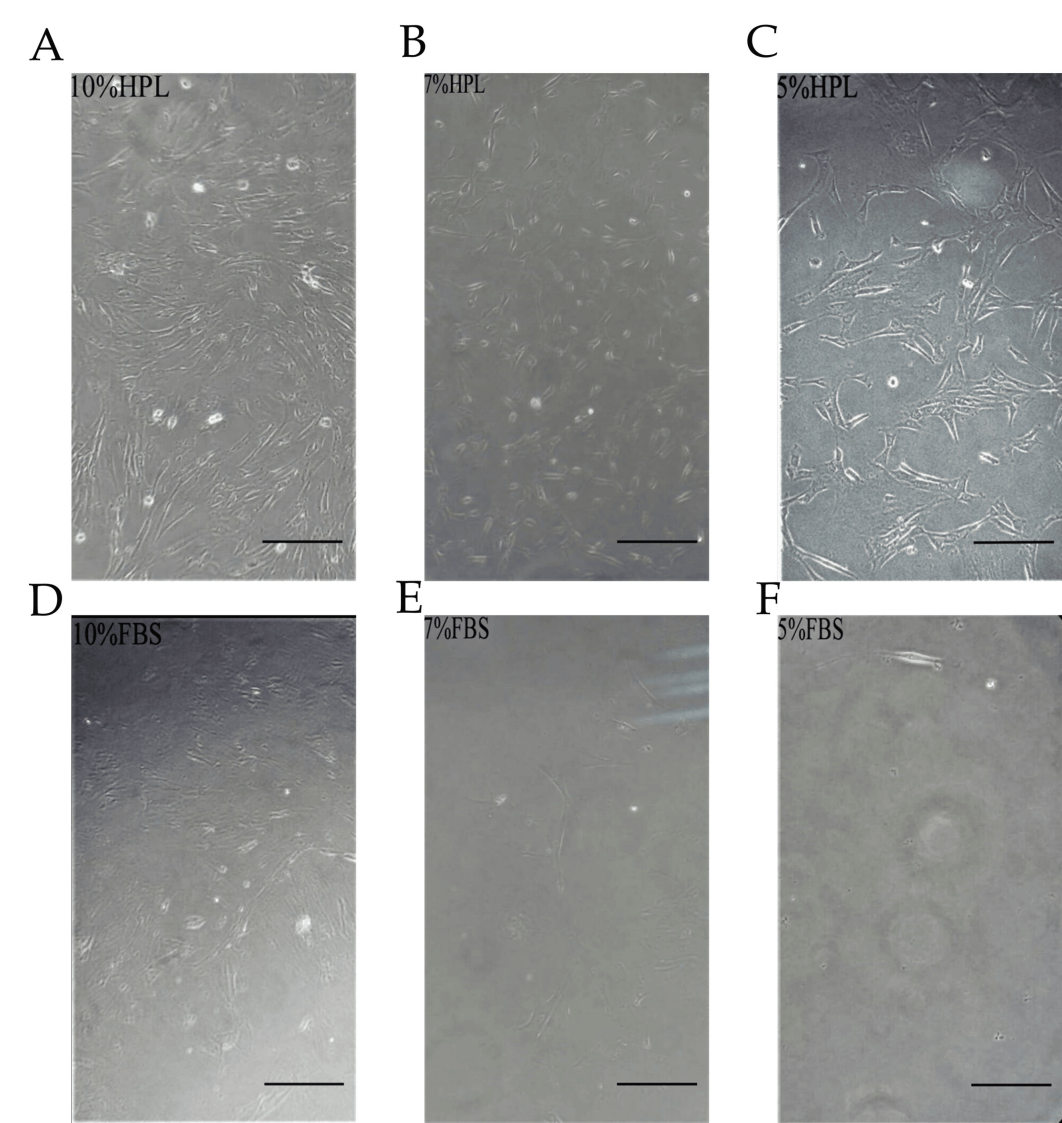
Different concentrations of HPL and FBS excreted dose-dependent growth-promoting activity After 18 days of WJ-MSC culture in DMEM supplemented with (5%, 7%, and 10%) of HPL and FBS, 10% HPL (A), 7% HPL (B), 5% HPL (C), 10% FBS (D), 7% FBS (E), and 5% FBS (F). Additionally, 10% HPL exhibited the highest number of MSCs and almost reached 90-100% confluence; confluence decreased gradually in 7% HPL, 10% FBS, and 5% HPL; and the 7% and 5% FBS showed very few fibroblast-like cells. Scale bar = 100 µm.

After three to seven days of explant culture, many cells migrated and adhered to the plastic surface with the ability to proliferate and expand to 80-90% confluence known as passage zero (P0). Variation was evident in the morphology of isolated WJ-MSCs between HPL and FBS-containing media. It also demonstrates that cells in HPL exhibited a shiny, small, rounded morphology, whereas, in FBS, they were larger and more heterogeneous in shape. Adherent cells isolated from WJ and cultured in either HPL or FBS maintained the same ability to form colonies but differed in size. Furthermore, it was demonstrated that CFU-F in HPL media was slightly larger and contained more cells than in an FBS culture. Cells also soon acquired the characteristic MSC morphology of being fusiform or spindle (fibroblast-like) shaped. This morphology was observed in 10% HPL (< three days), 7% HPL (five days), 10% FBS, and 5% HPL (seven days). It was also noticed that MSCs cultured in FBS presented a coarser, more flattened, and longer fibroblastoid profile, while MSC morphology in HPL was in more organized colonies and individually smaller, thinner, shinier, and with a more pronounced spindle shape and in more organized colonies (Figure 3). Moreover, we observed that different concentrations of HPL or FBS (5%, 7%, and 10%) had a dose-dependent growth-promoting activity effect on MSC culture. The proliferation rate in 10% HPL was the highest, with cells reaching 80-90% confluence within 14 days, whereas in 7% HPL, 5% HPL, and 10% FBS the cells reached 80-90% within 16 and 19 days, respectively. MSCs cultured in 7% FBS and 5% FBS did not reach 80% confluence until 19 days (Figure 4).

UCB-Derived MSC

MSCs were isolated from nine UCB units, each unit of about 40 mL processed within five to eight hours. MSC morphologies among different concentrations of HPL and FBS were then observed. In HPL media, cells appeared bright, small, and rounded in shape, while FBS cells were altogether less pronounced in shape and differed more in size. Black dots were also noticed in some samples. Cells in HPL also colonized better than in FBS, with no colonies apparent in 5% of FBS.

**Figure 5:**
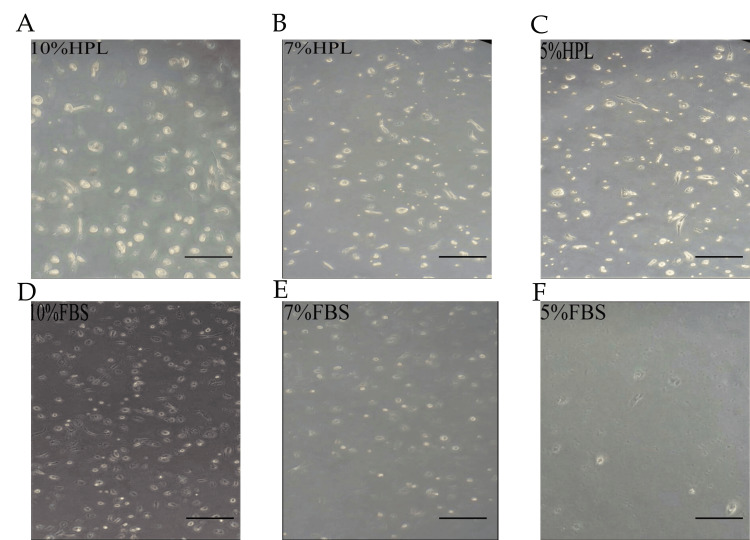
The impact of HPL and FBS on the growth of fibroblast-like cells Group B, samples day 14: The amount of fibroblast-like cells depended on the concentration of HPL or FBS: 10% HPL (A), 7% HPL (B), 5% HPL (C), 10% FBS (D), 7% FBS (E), and 5% FBS (F). We observed that the 10% HPL exhibited the highest concentration of fibroblast-like cells; it decreased gradually in 7% HPL, 10% FBS, 5% HPL, and 7% FBS, while 5% FBS did not show any fibroblast-like cells. Scale bar = 100 µm.

After eight days, cell proliferation was significantly different among samples, with results categorized into three groups (A, B, and C) according to sample morphology and proliferation rate. Group A comprised two samples that proliferated very slowly and showed no MSC expansion in all different media until day 19, while Groups B and C showed much proliferation. Between 12 and 17 days of culture, the adhered cells in Group B samples (three samples) started to show few fibroblast-like cells, with the number differing among various media. Additionally, 10% HPL media had the highest presence of spindle cells, and then it decreased gradually in 7% HPL, 10% FBS, 5% HPL, and 7% FBS, with no appearance in 5% FBS cells remaining in round shape (Figure 5). Then, the cells expanded until reaching 80-90% confluence between 16 and 22 days of culture among various media.

On the other hand, Group C (four samples) expanded slowly but had a high number of oval cells, especially in 10% FBS and all HPL-supplemented media. A fibroblast-like profile was absent in all media, with cells attaining 80-90% confluence between 21 and 26 days.

The results demonstrate that HPL boosted the adherence and proliferation of UCB-MSCs compared with FBS, and cells reached 80-90% confluence earlier (i.e., approx. 16 days in 10% HPL, 18 days in 7% HPL, 21 days in 5% HPL, and 10% FBS), while 5% and 7% FBS failed to reach confluence until day 26.

The adhesion capacity for MSCs cultured in HPL was also higher than that of FBS. Cells cultured in FBS medium detached from the plastic surface after three to five minutes of enzymatic action, whereas cells cultured in HPL, specifically cells cultured with 10% HPL, required additional trypsinization (twice added trypsin).

Viability Count of MSCs

**Figure 6:**
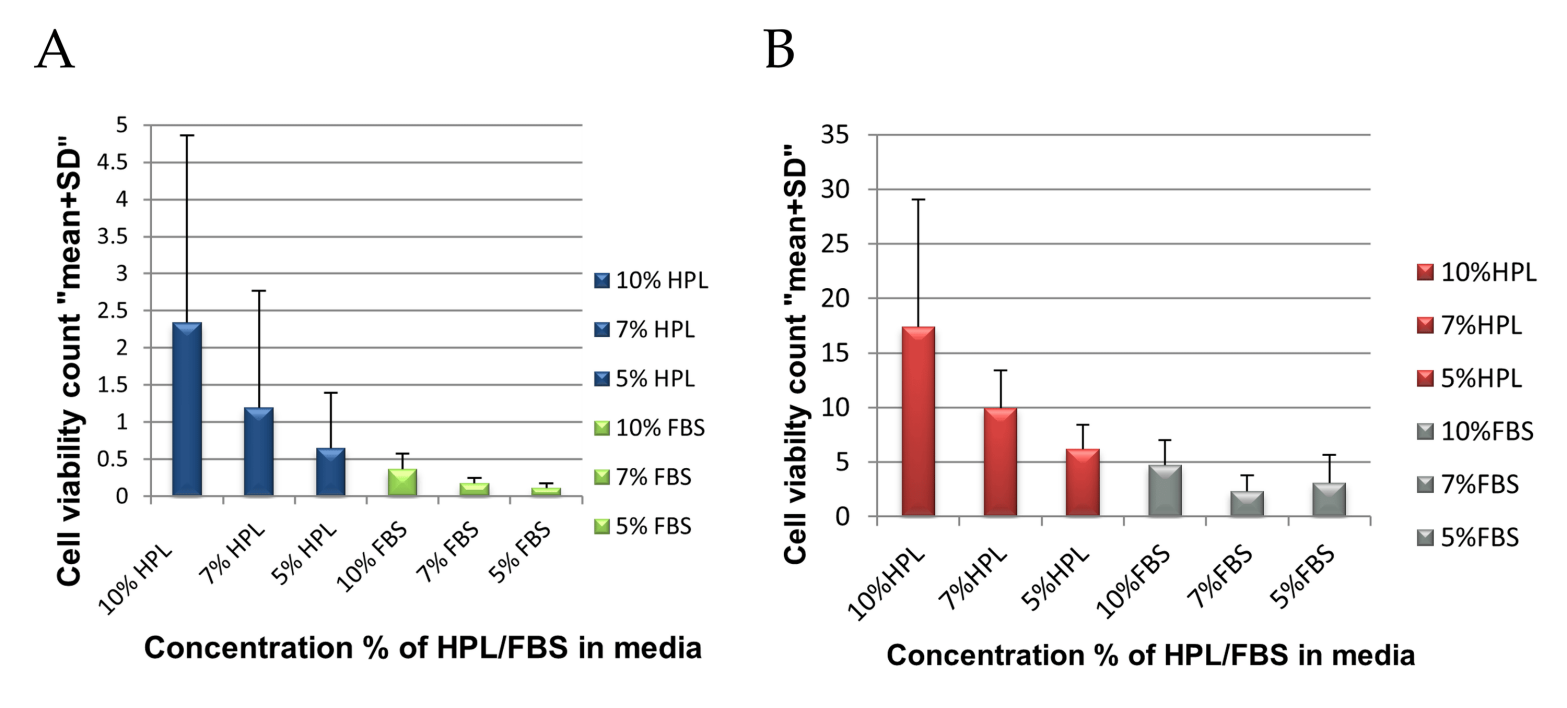
Viability count of MSCs The effect of different concentrations of HPL and FBS (5%, 7%, and 10%) on the viability count of MSCs isolated from Wharton's jelly was shown in (A) and cord blood in (B) at the end of P0. The results of viable cells are presented as mean. The ANOVA test analysis was used to analyze the results of this experiment (p-value 0.002).

Cells were collected through enzymatic dissociation using a trypsin-EDTA solution. This occurred at 19 days for the WJ-MSCs culture and between 22 and 26 days for the CB-MSC culture. In each case, viable cells were counted to assess UCB and WJ-MSC expansion according to the culture medium under six different concentrations (5%, 7%, and 10% of HPL and the same for FBS). Our results revealed that MSC viability count was significantly higher when they were cultured in media supplemented with HPL than in media supplemented with FBS in both cord blood and WJ-MSCs (p-value=0.002), as shown in Figure 6. When comparing the effect of different concentrations of HPL or FBS (5%, 7%, and 10%) on MSC viability count, we found that 10% HPL gave the highest count and that 7% and 5% HPL gave more reliable results than 10% FBS, but this difference was not statistically significant. On the other hand, there was a significant decrease in viable cell count from 7% and 5% FBS cultures.

Immunophenotypic characterization of UC-MSCs: flow cytometry analysis

**Figure 7:**
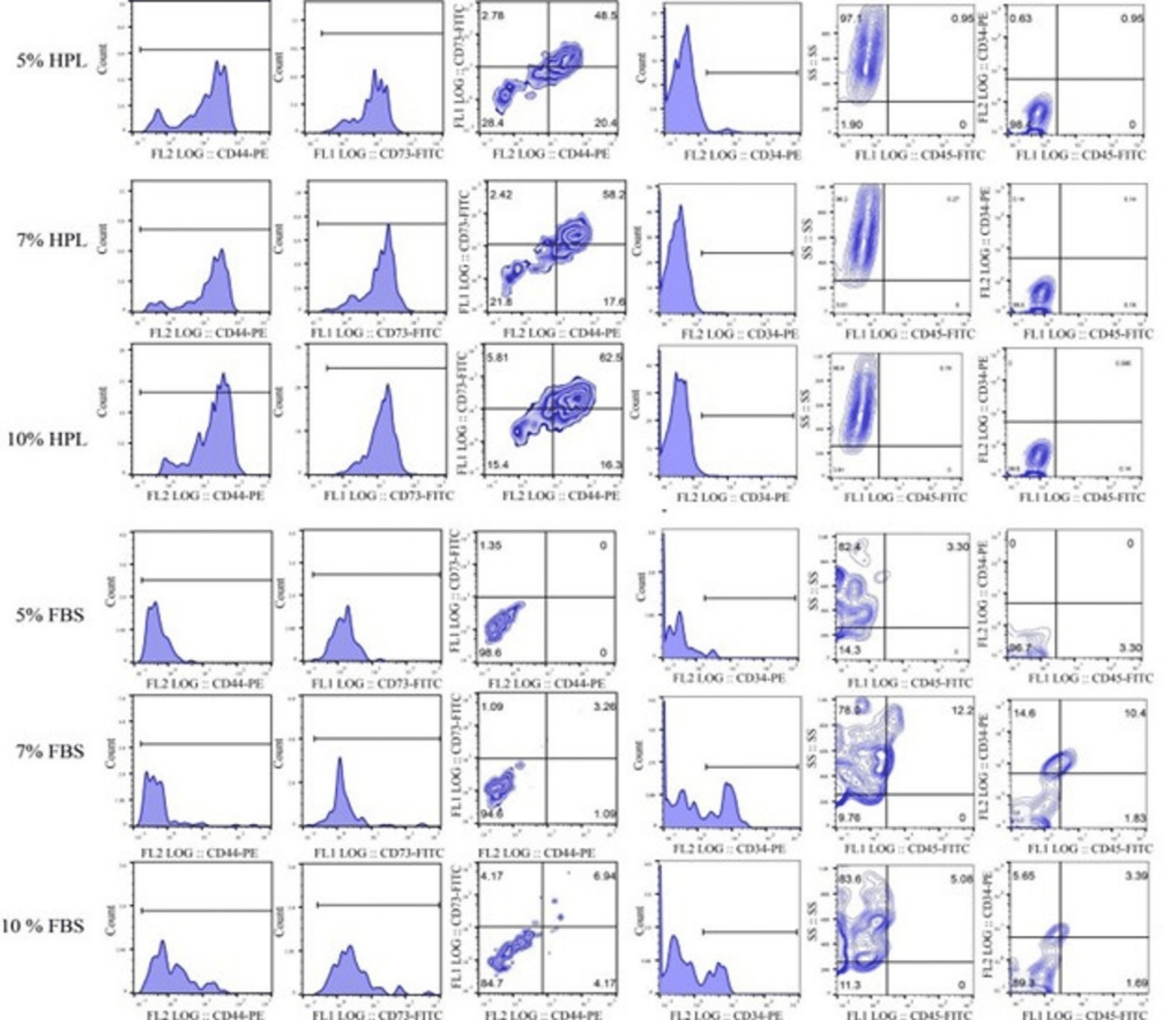
Characterization of membrane antigen expression of WJ-MSCs cultured at passage 0 in six different media The histogram plots represent the flow cytometry analysis of the umbilical cord MSCs cultured in media supplemented with HPL (10%, 7%, and 5%) and FBS (10%, 7%, and 5%) with directly labeled monoclonal antibodies against MSCs and hematopoietic markers CD44, CD73, CD34, and CD45, respectively. MSCs cultured in HPL were negative for hematopoietic markers CD45 and CD34 but were positive for CD73 and CD44. Additionally, 10% HPL expresses higher positive markers, whereas MSCs cultured in FBS were negative for hematopoietic markers CD45 and CD34. Most of the cells cultured in 5% FBS and 7% FBS were negative for CD44 and CD73, but cells cultured in 10% FBS showed higher positivity for CD44 and CD73.

Results showed that umbilical cord-derived MSCs (UCB-MSCs and WJ-MSCs) in all media were negative for the hematopoietic markers CD34 and CD45. WJ-MSCs and UCB-MSCs cultured in HPL were positive for CD73 and CD44; the strong positivity was most evident in 7% and 10% HPL concentrations. Meanwhile, most of the WJ-MSCs and UCB-MSCs cultured in FBS, especially 5% FBS and 7% FBS, were negative for CD44 and CD73, but cells cultured in 10% FBS showed higher positivity for CD44 and CD73. Our data showed a significant difference between the six culture conditions in the expression level of positive and negative markers (Figure 7).

## Discussion

The human umbilical cord (HUC) has recently been introduced as a promising source of MSCs, and it has gained great attraction due to its availability and the non-invasive nature of the collection. Furthermore, access to UC-MSCs is not encumbered with ethical challenges. The purpose of this study was to isolate and expand MSCs from UCB and WJ under xenogenic-free conditions to develop an efficient, safe, and cost-effective protocol for clinical application and to assess and compare the expanded MSCs in culture media supplemented with different concentrations of FBS or HPL. According to Arutyunyan et al. in 2016 [30], this study demonstrated 100% efficiency in isolating MSCs from WJ.

Traditionally, several protocols and culture methods have been developed for the isolation of MSCs from WJ with the most common two methods being explant culture and enzymatic digestion. Hendijani et al. [13] demonstrated a preference for the explants method but found that the protocol took longer. Beeravolu et al. additionally reported that partially digested prenatal tissue explants by trypsin solution with human serum as a supplement in culture media produced cell outgrowth without causing excessive damage to the cells, preserved viability, and generated a higher number of homogeneous populations of MSCs, whereas complete tissue digestion had poor yields of isolated cells [31]. Conversely, Bieback et al. indicated that the human platelet lysate supports the isolation and expansion of BM-MSCs in comparison to FBS. Platelet lysate was seen to be the premier component, assuring enriched cell numbers, cell identity, purity, sterility, immuno-phenotyping, and potency [21].

Based on these previous studies, the current study developed a protocol combining enzymatic and explant techniques using trypsin alone for partial digestion. Trypsin accelerates the rate of cell migration from tissue explant and decreases the cellular stress associated with complete enzymatic digestion. Moreover, the presences of tissue pieces mimic the in vivo environment by releasing cytokines and growth factors that stimulate cell growth. Additionally, this protocol added an advantage by preparing HPL using calcium gluconate and testing it in different concentrations as an alternative to FBS looking for a GMP-compliant method for the generation of allogeneic human platelets lysate for MSC cultivation. Our results showed that the cultivation media supplemented by HPL exhibited a vast MSC proliferation rate, resulting in bigger size colonies with a higher number of cells and distinct cell morphology, while the time for reaching ≥ 80 confluences is decreased when compared with media supplemented by FBS. Our findings are in agreement with Burnouf et al. in 2015 [23], Bernardo et al. in 2011 [8], and Doucet et al. in 2005 [25], which demonstrated that media supplemented with HPL enhanced MSC expansion and reduced the time to reach 80-90% confluence. Moreover, HPL was found to increase fibro-blastoid colony-forming unit (CFU-F), besides displaying pronounced MSC morphology in comparison with media supplemented with FBS [5].

In this study, we evaluated three different concentrations of HPL and compared them with FBS for the expansion of WJ-MSCs in vitro. Benchmarks used were MSC proliferation rate, morphology, viability, and the maintenance of their phenotype stability. Our data exhibited that 10% HPL is superior in terms of proliferation rate; therefore, the MSCs reached 80% confluence early (within 14 days) when compared with other media supplemented with 7% HPL, 5% HPL, and 10% FBS, which need 16 days and 19 days, respectively, to reach 80% confluence, while the 7% and 5% FBS did not reach the confluence for more than 19 days. Correspondingly, we noted that the spindle shape of MSCs appeared rapidly, especially in 10% HPL, leading to the formation of big CFU-F, which then decreased gradually in 7% HPL, 5% HPL, and 10% FBS. Mediums of 7% and 5% FBS had no discernible CFU-F presence, and accordingly, MSC presence was minimal. Furthermore, 10% HPL medium had a higher viability cell count when compared with other HPL concentrations and FBS medium. This suggests that an increase in HPL concentration provides a high amount of platelet granule factors that enhance MSC proliferation.

Although researchers have, for many years, isolated CB-MSCs, an argument still exists as to whether CB is a good source of MSCs for cell therapy or regenerative medicine. This may concern whether both the low frequency of MSCs in full-term CB and reliable culture conditions to isolate and expand UCB-MSCs have yet to be defined [32,33]. Contemporary research focused on the quality of CB unit selection by maximizing net volumes (more than 33 mL), ensuring no evidence of clotting and hemolysis, and limiting storage time to under 15 hours. Different culture protocols were also adopted using protein-coated plates involving the depletion of lymphocytes and monocytes from MNC before plating [34-37]. Cells were also cultured in hypoxic conditions, or cytokines were added to the primary culture medium [38]. Cumulatively, yields varied from 10% to as high as 60% [39], but by applying the aforementioned recommendations for CB unit selection and using HPL, we were able to standardize MSC isolation at 77.7%. In this study, we noted that UCB-MSCs expanded more slowly, requiring 16-26 days to reach 80-90% confluence compared to WJ-MSCs at 14-19 days. This finding agrees with Secunda et al., who found that WJ took 15 days to reach 80% confluence, while UCB required 23 days [39]. Isolated UCB-MSCs showed different types of morphology among the collected samples; most of them showed an oval shape and some exhibited fibroblast-like morphology, and the rest of them had weak expansion and did not proliferate until day 19 of culture. Similar results of differing morphology were obtained by Secco et al. in 2008 [40].

In the present study, we evaluated the effect of HPL on UCB-MSC proliferation and viability in P0 and then compared it with the effect of FBS. We noted that HPL in higher concentrations enhanced MSC growth, and we selected 10% HPL as the best concentration, both in terms of an increased proliferation rate and the highest viable cell count. Other concentrations of HPL (7% and 5%) gave comparable results to 10% FBS, but lower concentrations of FBS could not support the proliferation of MSCs. These results concur with the findings of Pham et al. in 2014 [41].

In this study, we confirmed that HPL can replace FBS for HUC-MSCs only according to morphological observation, proliferation, viability count, and expression of MSC CD markers. However, there are several limitations to acknowledge. First, we did not study the MSC differentiation properties, which is one of the properties mentioned by the International Society for Cell and Gene Therapy (ISCT) for MSC characterization. Further studies are required to examine this aspect to provide more validation of HPL as a substitute for FBS and assess its long-term use in clinical applications. Finally, while this study demonstrates the feasibility of using HPL for the in vitro expansion of HUC-MSCs, further studies are needed to develop validated HPL preparation protocols to ensure safety and minimize variability to translate it to clinical settings.

## Conclusions

MSCs were isolated from the UC (WJ and CB), which is considered an attractive source of MSCs due to its availability and can be easily collected without invasive procedures. Moreover, it is free from ethical concerns. The explant technique with partial enzymatic digestion is an effective protocol for isolating MSCs from WJ due to its ease of manipulation, short processing time, minimal cellular harm, and cost-effectiveness as it uses only trypsin for digestion. Human platelet lysate is a safe, powerful, and cost-effective alternative to FBS in the propagation and expansion of human MSCs, and 10% HPL had the best growth rate, highest viability count, and high expression level of CD-positive markers compared to other HPL concentrations. Furthermore, 5% HPL could replace 10% FBS, but 7% and 5% FBS are not good for MSC expansion. WJ-MSCs proliferated better than UCB-MSCs, which suggests that WJ is a more promising source for MSC isolation for clinical application.
